# Bitten by a Rat—Another Case

**Published:** 1872-02-15

**Authors:** M. Reece

**Affiliations:** Abingdon, Ill.


### BITTEN BY A RAT—ANOTHER CASE.

BY M. REECE, M. D., ABINGDON, ILL.

When reading Dr. Earle's case, reported in the first number of the Examiner for this year, I was reminded of several cases of a similar character I have had under my care, and especially of the following one, of which I kept the notes at the time, and which I simply transcribe. It will be observed that there is a considerable degree of resemblance in the general features of the two cases.

June 11, 1866.—Consulted by Mr. S. W., 38 years of age, by occupation a farmer, for a painful condition of the little finger of the right hand. Two weeks ago he received a bite from a rat he had caught by the back of the neck. The bite was through the skin, just behind the second joint. The wound healed, and nothing more was thought of it until four days ago, when it became hot, red, swollen and painful. It now presents the appearance of erysipelatous inflammation. The back of the hand is puffed and inflamed. Within a day or two he has been chilly, with headache, aching of the bones and back; pulse 115; tongue coated; skin dry and hot; eyes red and injected; is in great pain, which commences at the sore on the finger, and extends upwards to the axilla; says he thinks his mind is becoming affected by the agony he is in, and cannot remember certain things he is told to do.

Is to take a cathartic at bedtime, and anodynes frequently enough to relieve pain. Tr. Iodine to be applied to the back of the hand and finger every four hours, and a poultice of Indian meal to envelope the whole hand. Also to take tincture of iron and quinine every four hours. (Quin. sul. ʒ iss., tr. ferri chl. f ℥ i. M. Dose 15 drops.)

12th. Bowels moved last night; has suffered great pain; the affected parts are swollen to a greater extent than yesterday. To take anodynes more freely, and continue treatment.

13th. Still suffering intense pain; pulse 130; finger and hand continue to swell; to-day made an incision into the little finger; a drachm of yellow serum escaped; the incision gave no pain whatever. To continue treatment.

14th. Was sent for in great haste; patient suffering intensely; great difficulty in swallowing; soreness about the muscles of the lower jaw; pain along the back and in the muscles of the thighs; is very apprehensive of some impending danger; has an undefined fear of something he cannot describe; there are many symptoms of tetanus. Applied cloths, moistened with chloroform, to the spine, a blister to the nape of the neck, and gave morphine freely.

15th. Found the patient much better. A mass of dead tissue around the bite, extending in depth to the bone, has almost sloughed away; removed it with dressing forceps and scissors, leaving healthy, granulating edges; the swelling of the hand is beginning to decrease; for the past twenty-four hours has been bathed in a profuse and drenching perspiration.

18th. Again sent for to see the patient. Found him in a condition similar to what he was on the 14th; employed the same means as on the former occasion; the sore on the finger improving.

19th. Better than yesterday, but still bathed in profuse and exhausting perspiration; complains to-day of pain in the axilla; some red points appear over the course of the veins of the fore-arm, over which tincture of iodine is to be applied; to take anodynes when found necessary.

22d. Still has pain extended upwards along the fore-arm and arm; the glands in the axilla considerably swollen and tender; tincture of iodine to be applied freely from the hand to the shoulder, to the swollen glands, and all enveloped in a poultice; feels very weak; exhausting perspiration continues. In addition to quinine and iron is to have wine freely.

30th. An abscess has formed in the right axilla, which I opened to-day. It discharged two ounces of pus; complains of great pain in the chest; it hurts him to take a deep inspiration; has no desire for food whatever; the place where the bite was made is nearly healed.

It was not until about the middle of July, or more than a month after his attack, that he was able to walk around, and then he was in a very weak and nervous condition, and was very easily exhausted. The sore on the finger healed entirely within two weeks from the time he was taken sick. A great many small pustules appeared along the arm for a few days, and then gradually disappeared.

A prominent symptom, after the subsidence of the more severe constitutional symptoms, was a pain in the chest, beneath each breast; it was believed to be chiefly muscular; this pain was greatly increased upon the slightest exertion; there was always great lassitude in mind and body. He was advised to ride about and take as much fresh air as possible. Being a man of great energy he attempted to to follow the advice, but at first had to lie upon a bed in a wagon, so great was the prostration. By degrees, however, he gradually but slowly gained strength so that he could ride in a buggy, and then on horseback.

Six months afterwards (from the date of his attack) the condition of Mr. W. was as follows: The effects of the bite were still visible in the impaired use of his right hand, and still further in his inability to resume the active duties of his occupation; he was unable to walk rapidly without becoming breathless, and causing violent palpitation of the heart, and his strength so much impaired that he could only lift the lightest weights.

In addition to the above case I will, as briefly as possible, report three others:

B., a young woman, in trying to drive a pig out of her yard, caught it by the ear, when the animal, becoming infuriated, got her hand in its mouth, slightly crushing two of the fingers of the right hand. The fingers were not seriously hurt, but in the course of a week they became badly inflamed. The inflammation extended up the hand and wrist, and was accompanied by severe constitutional disturbance, extending over a period of two weeks.

Mrs. C., in attempting to put a pet squirrel into its cage, was bitten by the enraged animal on the back of the right index finger. Within twenty-four hours there was intense pain in the whole hand and fore-arm, with chills and fever. The hand swelled to a fearful extent; abscesses formed in different parts of it, and the tissues of the bitten finger sloughed entirely. Portions of the flesh on the back of the hand also sloughed out, exposing the tendons of the muscles in that region. Her recovery was long and tedious, and she never perfectly recovered the use of her hand. Two fingers became entirely stiff and useless, and two years ago, upon receiving an injury to the finger that was bitten, she had it amputated, as it was a useless and even annoying appendage.

Mrs. G. left her infant of ten months asleep in the cradle. Hearing it crying she returned to the room, and as she entered the door saw a rat jump from the cradle near the child's head. Thinking the child had only been frightened by the rat, she had no thought of its having been bitten by it. In the course of a few days, however, the child became exceedingly sick. It was feverish, and at a point near the left shoulder there was an erysipelatous blush which extended upwards along the side of the neck. Moreover, near the center of this spot could be seen two little abrasions, each filled with a drop of yellow serum.

The inflammation extended over the whole body of the child; first to the neck, face and head, then downwards to the chest, abdomen and inferior extremities. It became badly swollen, and in many parts blisters appeared on the skin. The fluid from the blisters was of a yellowish color, and left a stain upon the clothes it was impossible to wash out.

The child, contrary to the expectation of every one who saw it, after remaining in the condition above described for five days, made an excellent and rapid recovery.

